# Introducing the Intergovernmental Policy Output Dataset (IPOD)

**DOI:** 10.1007/s11558-023-09492-6

**Published:** 2023-06-06

**Authors:** Magnus Lundgren, Theresa Squatrito, Thomas Sommerer, Jonas Tallberg

**Affiliations:** 1 University of Gothenburg, Gothenburg, Sweden; 2 London School of Economics, London, UK; 3 Universität Potsdam, Potsdam, Germany; 4 Stockholm University, Stockholm, Sweden

**Keywords:** International organizations, Policy, Policy agendas, Decision-making

## Abstract

There is a growing recognition that international organizations (IOs) formulate and adopt policy in a wide range of areas. IOs have emerged as key venues for states seeking joint solutions to contemporary challenges such as climate change or COVID-19, and to establish frameworks to bolster trade, development, security, and more. In this capacity, IOs produce both extraordinary and routine policy output with a multitude of purposes, ranging from policies of historic significance like admitting new members to the more mundane tasks of administering IO staff. This article introduces the Intergovernmental Policy Output Dataset (IPOD), which covers close to 37,000 individual policy acts of 13 multi-issue IOs in the 1980–2015 period. The dataset fills a gap in the growing body of literature on the comparative study of IOs, providing researchers with a fine-grained perspective on the structure of IO policy output and data for comparisons across time, policy areas, and organizations. This article describes the construction and coverage of the dataset and identifies key temporal and cross-sectional patterns revealed by the data. In a concise illustration of the dataset’s utility, we apply models of punctuated equilibria in a comparative study of the relationship between institutional features and broad policy agenda dynamics. Overall, the Intergovernmental Policy Output Dataset offers a unique resource for researchers to analyze IO policy output in a granular manner and to explore questions of responsiveness, performance, and legitimacy of IOs.

**Supplementary Information:**

The online version contains supplementary material available at 10.1007/s11558-023-09492-6.

While the role and impact of international organizations (IOs) remains debated in international relations (IR) (Abbott & Snidal, 1998; Barnett & Finnemore, 2004; Gruber, 2000), there is a growing recognition that they formulate and adopt policy in a wide range of areas (Hooghe et al., 2017; Zürn et al., 2021). IOs have emerged as key venues for states seeking joint solutions to challenges such as climate change or epidemics, and to establish frameworks to bolster trade, security, and development. In recent years, many of the most consequential intergovernmental policy agreements have been formulated and adopted within the framework of IOs, such as the 2015 Paris Agreement on climate change (United Nations), the 2007 African Charter on non-acceptance of unconstitutional changes of government (African Union), and the 2020 Next Generation EU recovery fund to support countries in the aftermath of COVID-19 (European Union). Next to such historic decisions, IOs continue to churn out routine policy output with a multitude of purposes, ranging from updating trade quotas to administering pensions for their staff.

As scholarship on the role of IOs in global governance has expanded, systematic data collection efforts have intensified. In recent years, researchers have introduced datasets on important IO characteristics, such as their membership (Pevehouse et al., 2020), openness to transnational actors (Tallberg et al., 2013), authority (Hooghe et al., 2017; Zürn et al., 2021), and overlap (Haftel & Lenz, 2022). These efforts share an emphasis on comparative analysis, employing standardized measures across organizations to capture patterns of variation and similarity. In contrast, data collection on IO policy output remains focused on single policy fields (e.g., Rutkowski, 2007) or individual organizations or bodies (e.g., Alexandrova et al., 2014; Cockayne et al., 2010; Frederking & Patane, 2017). While these datasets are important resources – as well as a source of inspiration for this project – their narrow focus makes them unsuitable for the type of broad, comparative analysis that is required to understand the patterns, sources, and consequences of intergovernmental policy.

In introducing the Intergovernmental Policy Output Dataset (IPOD), we seek to move beyond these limitations. Encompassing the policy output of 13 multi-issue IOs in the 1980–2015 period, the dataset covers 37,000 individual policy acts. The unit of analysis is the individual policy act, each of which is coded along several dimensions: topic (issue orientation), type (e.g., regulatory, distributive), instrument (legal bindingness), and target (addressee)). Recording these dimensions at the level of each policy act provides researchers with a fine-grained perspective on the structure of an IO’s policy output and allows for aggregation at other levels of analysis amenable to comparisons across time, policy areas, and organizations.

By providing a new source of systematic data on IO policy, the IPOD dataset can help address several debates and under-examined issues in the contemporary IR literature. First, broad, quantitative data on IO output can generate *descriptive insights* into larger patterns and trends across time, policy areas, and IOs. This article points to several examples of how such patterns can help adjudicate claims regarding the supposed decline of IOs (e.g., Debre & Dijkstra, 2021b), widening IO authority (e.g., Zürn, 2018), the role of IOs in international legalization (e.g., Abbott & Snidal, 2000), or expanding IO bureaucracies (e.g., Parizek & Stephen, 2021). Second, the presented dataset enables new comparative research on IO policy output as a *dependent variable*, including comparative studies of variation in decision-making performance, policy orientation, and bindingness. While research has taken some steps in this direction (e.g., Sommerer et al., 2022), wider advances in the explanatory study of IO policy and decision-making have been held back by the lack of comparative data. Finally, IPOD opens up for advancing our understanding of IO policy output as an *independent variable*. Comparative and highly granular data will provide additional leverage in tackling questions relating to IO performance (Gutner & Thompson, 2010; Lall, 2017), the legitimacy of IOs, and the distributive consequences of IOs (Fehl & Freistein, 2020).

The rest of this article is structured into five parts. In the following section, we place the dataset in context, summarize existing data sources, and motivate why a new dataset is required. We then describe the creation of the dataset, discussing sample selection, data collection, and coding procedures. The third section provides a brief overview of the data, outlining temporal and cross-sectional patterns for the key variables. The fourth section provides a concise illustration of the utility of the data, applying models of punctuated equilibria in a comparative study of the relationship between institutional features and agenda dynamics, typically viewed as one facet of IO responsiveness. The final section concludes and identifies how the IPOD data can be used to advance several research agendas in IR.

## Existing data on IO policy

Seeking to map and understand the ability of political institutions to arrive at decisions, scholars have collected a rich body of quantitative data on various forms of policy output. In light of the longer traditions of comparative politics and policy studies, it is natural that the most expansive data sources relate to political institutions at the national level. For example, researchers associated with the Comparative Agendas Project have generated large-scale datasets on the legislative output in several countries, including the United States (Adler & Wilkerson, 2013), Denmark (Green-Pedersen & Mortensen, 2010), and Germany (Breunig & Schnatterer, 2019). Other researchers have collected cross-national data on specific policies, such as COVID-19 government responses (Hale et al., 2020) or welfare entitlements (Scruggs et al., 2017). These sources provide a rich variety of data for comparative policy analysis.

In contrast, scholars interested in international policy output operate in a research environment with considerably fewer quantitative data sources. Existing data sources can be categorized into three main types.

The first type are sources that provide data on single policy areas within single IOs. While most research in this category relies on case-based methods, there is a growing number of studies that have collected quantitative data to help track policy output within a single organizational context. Nielson and Tierney (2003), for example, use quantitative data to trace environmental policy reform within the World Bank (WB). Others have compiled data on the output of United Nations (UN) human rights treaty bodies (Kahn-Nisser, 2018), the UN Human Rights Council (Terman & Voeten, 2017), and the UN Security Council’s decisions on UN peacekeeping mandates (Di Salvatore et al., 2022).

The second type of sources provide cross-IO data on single or a small number of policies. For example, Rutkowski (2007) compares the educational policies promoted by a sample of IOs, Gallagher and Yuan (2017) present comparative data on sustainable development policies across eleven regional development banks, while Tallberg et al. (2020) map the adoption of eight liberal policies across 18 multi-issue IOs.

A third type provides comprehensive data on the policy decisions by single IOs. Focusing on the EU, Alexandrova et al. (2014) presents data on the topics covered in the policy agenda of the European Council from 1975 to 2012, while Häge (2011) provides observations on 29,000 EU legislative processes between 1975 and 2009. Other EU-oriented efforts, focused on decision-making rather than policy characteristics, include the Decision-Making in the European Union (DEU) I-III datasets (e.g., Arregui & Perarnaud, 2022) and Alesina et al. (2005). Next to the EU, there are several datasets relating to the policy output of the UN. Both Cockayne et al. (2010) and Frederking and Patane (2017) compile detailed data on Security Council Resolutions, covering the time periods 1989–2003 and 1989–2013, respectively (see also Lundgren & Klamberg, 2022; Allen & Yuen, 2020; Beardsley et al., 2017). Other researchers have compiled data on International Monetary Fund (IMF) and WB funding decisions (e.g., Dreher et al., 2009; Copelovitch, 2010).^1^

The data sources available for researching intergovernmental decision-making and policy have provided a crucial foundation for advancing the scientific understanding of global and regional governance. However, these sources suffer from two main limitations. First, existing data sources tend to focus on individual IOs, specific policy areas, or both. This tendency could be unproblematic if different datasets relied on common or comparable approaches, measures, and time frames, which would allow for mergers across datasets. However, except for the Comparative Agendas Project (which only covers the EU among international organizations), there is little uniformity with regard to conceptualizations or coding practices.

Second, current datasets place a heavy emphasis on policy content, privileging information on what an agreement is about over other features, like its bindingness or targeted audience.^2^ This focus on content is likely a consequence of long-standing research interests in the policy literature, the fact that policy content is relatively easy to detect, and the influence of research programs such as the Comparative Agendas Project. While policy content is a key feature, limiting data to this sole aspect constrains the range of questions that we can ask about intergovernmental policy.

Taken together, these shortcomings imply that existing data sources on intergovernmental policy are too limited and disjointed to provide a sufficient evidentiary basis for researchers interested in systematic comparison across IOs, time, and features of policy design.

## Creating a new dataset: scope, coding, and variables

### Scope

We focus our dataset on policy produced by IOs, defined as intergovernmental, multilateral, and bureaucratic organizational structures established to further cooperation among states (Martin & Simmons, 2012; Pevehouse et al., 2020). A common conceptualization of IOs requires them to have at least three member states and possess a permanent secretariat or similar signs of institutionalization (Pevehouse et al., 2020). We adopt this understanding. The COW-IGO 3.0 dataset lists 451 unique IOs, of which between 230 and 286 were active between 1980 and 2014 (Pevehouse et al., 2020). From these organizations, 53 can be classified as having a multi-issue orientation defined as having a mandate that enables them to formulate policies in at least three different substantive areas (cf. Tallberg et al., 2020).^3^ These multi-issue IOs represent the population of interest for our study. In contrast with task-specific or single-issue IOs, which operate in one narrow, often technical policy field, multi-issue IOs have broader and more flexible mandates, situating them at the center of the global governance architecture. Given their broad mandates, multi-issue IOs are particularly suitable for examining how global priorities vary over time, as reflected in shifts in policy orientation. While there are some task-specific IOs that maintain a dominant presence in global governance, such as the IMF or the World Bank, their core mandate relates to one or a small set of issue areas and, unlike multi-issue IOs such as the UN or AU, they are not typically viewed as fora that states turn to for general deliberation and policy-making. Informal groupings of countries, such as G-7 or G-20, are excluded because they do not have the necessary bureaucratic structure to qualify as an IO according to our above definition.

We limit our selection to organizations included in the widely used Measuring International Authority (MIA) dataset on the authority and policy scope of IOs, which includes 29 organizations with core mandates in three or more issue areas (Hooghe et al., 2019).^4^ From the MIA sample, we selected 13 organizations for inclusion in our dataset (Table 1), based on two key considerations. First, the sample was selected to attain a wide geographic scope (Fig. A1 and Table A1 in the Online Appendix). It includes the key global IO (UN) and important multi-issue organizations from all regions of the world. Most of the IOs in our sample classify as the primary multi-issue organization in their region or sub-region. Including IOs with diverse regional origins and coverage avoids problems of geographic bias that has been identified in previous research on IOs and IR (cf. Tickner & Wæver, 2009).

**Table 1:** IPOD sample

IO	Region	Years	Decision-making body	Primary policy formats
Association of Southeast Asian Nations (ASEAN)	Asia–Pacific	1980–2015	Ministerial meetings	Communiqués
Organization of African Unity / African Union (AU)	Africa	1980–2015	Assembly of the African Union	Resolutions, Decisions, Declarations
Andean Community (CAN)	Americas	1980–2015	Commission	Decisions
Caribbean Community (CARICOM)	Americas	1980–2015	Ministerial councils	Communiqués, Declarations
Commonwealth of Nations (COMW)	Global	1980–2008	Heads of Government Meetings	Communiqués
European Union (EU)	Europe	1980–2015	Council of the European Union	Directives, Regulations, Decisions
Nordic Council (NC)	Europe	1980–2015	Council of Ministers	Proposals
Organization of American States (OAS)	Americas	1980–2015	General Assembly	Resolutions, Declarations
Organization of Islamic Cooperation (OIC)	Global	1980–2015	Ministerial councils	Resolutions
Pacific Islands Forum (PIF)	Asia–Pacific	1980–2015	Heads of State and Government	Communiqués
South African Development Community (SADC)	Africa	1980–2015	Summit of Heads of States or Government	Communiqués, Declarations
Shanghai Cooperation Organization (SCO)	Asia–Pacific	2002–2015	Council of Heads of State	Communiqués
United Nations (UN)	Global	1980–2015	Security Council	Resolutions, Presidential statements

Second, the IOs in our sample represent relevant subgroups of the larger population of multi-issue IOs in terms of membership size and institutionalization. Our sample includes organizations with small, medium, and large memberships (Table A2). The mean number of member states of IOs in our sample is 23.7, very close to the mean of 24.2 for the multi-issue organizations listed in the COW-IGO dataset (Pevehouse et al., 2020). With regard to institutional design, the selection in our data captures both weakly and strongly institutionalized organizations (Table A2). In terms of delegation, i.e., the extent to which IO bodies are independent of member states, the IOs in our sample have a mean score of 0.27 compared with 0.23 for the multi-issue IOs included in MIA dataset. A measure of “pooling,” the extent to which states employ collectivized decision-making and have ceded the national veto, come in at 0.31 for our sample against 0.23 for the MIA IOs (see also Fig. A2). Difference in means tests between our sample and the MIA sample of multi-issue IOs are not statistically significant with regard to membership, delegation, and pooling.

Overall, while we make no claims to perfectly represent the wider population of IOs, our sample reflects important characteristics of the relevant population, multi-issue IOs, with reasonable accuracy.^5^ Our selection of IOs represents approximately of 25% of recognized formal IOs with a multi-issue orientation. This is a significant expansion on previous studies on IO output which typically only cover one single IO. The ambitious coverage of this data is reflected in the time spanning 36 years and equaling nearly 37,000 policy acts.

In each IO, we focus on policy acts adopted by the principal intergovernmental decision-making body, such as the Council in the EU or the General Assembly of the Organization of American States (OAS) (Table 1).^6^ While the name, meeting frequency, and exact role of these bodies varies across IOs, we have selected them because they are similarly situated as the top intergovernmental decision-making organ of their respective organization. They establish its core policy direction and adopt strategic and operative decisions on issues pertaining to the IO’s mandate. Because they are the closest approximation to the domestic notion of a legislature and often make political commitments on behalf of the IO, they are a suitable place to study an IO’s overall policy output.

We focus on the years 1980–2015 because this period has seen tremendous growth in the scope and variability of global governance arrangements (Hooghe et al., 2017; Pevehouse et al., 2020).^7^ Covering 36 years, the data enable the construction of independent or dependent variables with significant longitudinal variation. They cover parts of the Cold War period (1980–1991), when many IOs were marked by the direct or indirect rivalry between the superpowers, the liberal reawakening after the end of the Cold War, when many looked to international organizations as central tools for promoting global public goods, and the accelerating contestation of global governance in play since the early 2000s, when the role and legitimacy of international organizations have become questions of debate and contestation.

### Data collection and coding

We collected information on all policy acts adopted by the selected intergovernmental decision-making bodies. In many cases, these bodies adopt different types of acts, such as resolutions, directives, declarations, decisions, and statements. We capture all significant types, following the nomenclature stipulated by each IO. Some of the decision-making bodies do not publicize individual acts but provide a summary of decisions adopted in a meeting of the main decision-making body, typically in the form of communiqués. In order to make this output comparable to that of other decision-making bodies, we disaggregated such summaries into individual policy acts.

We collected policy outputs via two main sources. First, for the majority of IOs, we were able to identify comprehensive online archives recording decisions by year and type. For example, the UN records all the resolutions and presidential statements adopted by the Security Council in chronological lists, with links to agreement texts. Second, where digital archives were incomplete or not available, we attained physical copies of policy acts via archival research and secondary sources. For example, the Nordic Council of Ministers did not offer an online archive, but its decisions were available in printed format in library series.

A multilingual team of nine trained research assistants hand-coded each act based on manual content analysis. All coding was based on a joint codebook with detailed coding rules for how to assign codes to each act across the main variables (see online appendix). To improve the quality of the coding, we established an online forum where coders could ask questions and exchange views, while the project team monitored the progress and quality of coding. When coding issues emerged, they were examined and resolved in coordination with the project leaders. In total, 36,987 acts were coded.

At two instances during the coding process, we carried out reliability checks. The first reliability check was undertaken at the pilot stage to identify potential shortcomings in the coding procedures. It indicated that intercoder agreement varied across variables and IOs but that only marginal modifications to the codebook were necessary. The second reliability check to assess intercoder agreement was undertaken once the coding was nearly complete, providing a better reliability estimate. Based on a test in which three coders independently coded a random sample of acts, drawn from the entirety of the dataset, average intercoder agreement was 81 percent. Fleiss’ kappa, a measure that considers the number of categories coded, varied between 0.62 and 0.70 for the key variables, indicating substantial agreement.

### Variables

Seeking to capture core dimensions of IO policy-making, the dataset records values on four key variables for each policy act: topic, type, instrument, and target. These are theoretically informed by existing conceptualizations of IO policy output (Tallberg et al., 2016) and the wider policy literature (e.g., Baumgartner et al., 2019) and resonate with policy dimensions used in comparative policy research (e.g., Fernandez-i-Marin et al., 2021). They represent features that are applicable to all policy-producing IOs, cover the most substantive aspects of policy output, and are central to many contemporary debates in IR. Differentiating acts along these four dimensions enables a rich, comparable, and fine-grained picture of IO policy output. The act-level variables can be aggregated to many other levels of analysis, such as the IO-year or IO-month, depending on the analyst’s interests.

**Policy topic** captures the thematic orientation of an act – the policy area with which it is concerned. As such, it provides a direct measure of the issues to which an IO directs its attention. Inspired by the Comparative Agendas Project (Baumgartner et al., 2019), we developed a two-tiered classification scheme for policy topics in global governance (see codebook). We distinguish 16 specific topics, such as economic development, security and defense, and IO governance, each of which is further divided into between 5 and 14 sub-topics. A key benefit of the two-tiered classification is that the data allow for analysis of IO attention to very specific issues (the sub-topics) as well as higher-level themes (the main topics). We code up to three main topics (with associated sub-topics) per act. For example, the Nordic Council (NC) 1998 agreement to develop the Nordic Environment Finance Corporation, an international finance institution aiming to promote environmental projects in Central and Eastern Europe, is coded as both “environment” and “economic development.”^8^

**Policy type** refers to the function of an act – what it seeks to do. Building on typologies developed for the study of domestic political systems (Almond & Powell, 1966; Lowi, 1972), we distinguish between five key types of policy: regulatory, distributive, declarative, constitutional, and administrative. A *regulatory* policy specifies actions that actors are either expected to take or refrain from taking, aiming to achieve desired interactions by addressing problems of coordination and collaboration. For example, a 2008 EU Directive concerning “road infrastructure safety management” is coded as regulatory, as it stipulates a set of rules and standards for member state policies. A *distributive* policy relates to the distribution or redistribution of goods and services among actors. For example, Resolution 36/12 of the Organization of Islamic Cooperation (OIC), adopted in 1981 and encouraging member states to assist Guinea with disaster relief, is coded as redistributive, since it promotes the reallocation of resources.^9^ A *declarative* policy asserts a joint position of the member states or the IO. It may be aspirational, assert agendas, or promote or condemn certain actions. For example, a Presidential Statement adopted by the UN Security Council in 2011, in which the main clause welcomes the news that “Osama bin Laden will never again be able to perpetrate acts of terrorism” is coded as declarative.^10^ A *constitutional* act stipulates rules that govern an IO or changes to its general organizational structure. For example, an act relating to “decision-making in the Caribbean Community,” summarized in a 1985 communiqué, is coded as constitutional.^11^ Finally, an *administrative* output concerns internal operational and managerial concerns of an IO. The decision to approve funding for the “construction of the SADC [South African Development Community] headquarters,” adopted by said organization in 2004, is coded as administrative.^12^

**Policy instrument** captures whether a decision creates legal obligations on the part of the signatories. In accordance with scholarship on legalization in international politics (Abbott & Snidal, 2000; Abbott et al., 2000), we distinguish acts as either legally binding (“hard law”) or non-binding (“soft law”). The distinction is informed by analysis of the provisions of each act, the competencies listed in each IO’s founding treaty, and relevant international legal interpretations. For example, UN Security Council Resolution 918, adopted in 1994 to impose an arms embargo against Rwanda, supervised by a sanctions committee, is coded as binding, whereas the Presidential Statement on Osama bin Laden referenced above, which did not generate legal obligations, is coded as non-binding.^13^

**Policy target** refers to the entity whose behavior or actions the policy is intended to influence of address. These are actors who are at the receiving end of the policy. We distinguish between three types of actors – IOs, states, and non-state actors – and whether the actors are associated with the policy-making IO or whether they are external. In total there are seven categories: IO, member states, selected member state(s), nonmember states, other IOs, and private actors. We categorize an act as targeting an IO if it relates to the IO’s supranational infrastructure, such as its secretariat, but as targeting member states if it relates to one or several states that are members of the IO. Private actors include corporations, non-governmental organizations, armed non-state actors, and individuals. We code up to three targets for each policy act. For example, a policy operating through member states but ultimately seeking to regulate commercial enterprises is coded as having two targets, member states and private actors.

Next to these four main variables, the dataset includes a number of identification variables, including *IO*, *year*, and *act title*. Each coded act is also given a unique *act identity number*.

## Patterns in the data: Policy across IOs and over time

To provide an overview of the data, the following figures present a series of descriptive patterns. We begin by illustrating the distribution of aggregated policy acts across IOs and over time, before continuing to explore patterns in the four main act-level variables of topic, type, instrument, and target. For each of these, we discuss key cross-sectional and longitudinal patterns based on descriptive plots and statistics. While we do not provide explanatory analysis of these patterns, we illustrate their relevance by discussing them in relation to research questions and ongoing debates in IR.

**Fig. 1:**
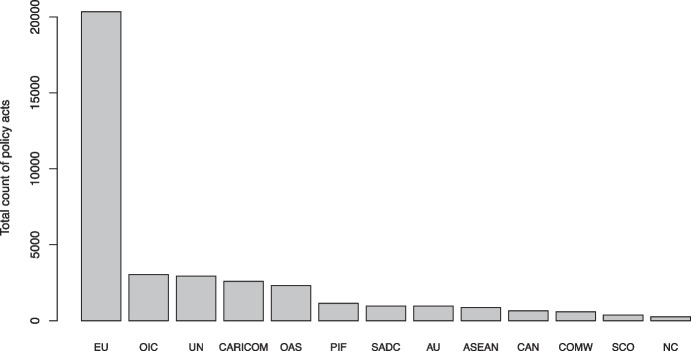
Total count of policy acts, 1980–2015, by IO

By aggregating act-level data to the IO- or year-level, we can attain insights into the broad patterns of IO policy output. As is clearly visible in Fig. 1, the 37,000 individual policy acts are unevenly distributed across IOs in the sample. Acts adopted by the EU represent a significant portion of the dataset, with a total policy volume exceeding that of the other twelve IOs taken together.^14^ Even among non-EU IOs there is considerable skew, suggesting that intergovernmental policy-making is concentrated to a smaller number of very active IOs, next to a large set of less active IOs. We note that several of the most active IOs, such as the OIC, the UN and OAS, have larger memberships, and that several of the less active ones, such as the Shanghai Cooperation Organization (SCO) and the NC, have fewer members.

**Fig. 2:**
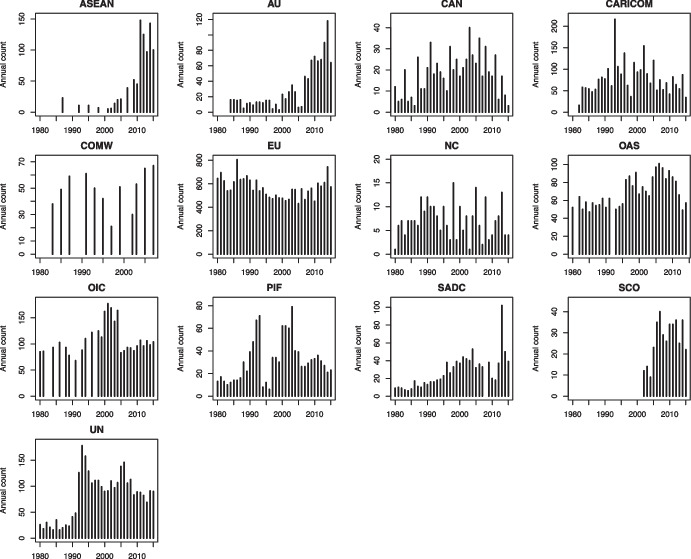
Annual count of policy acts, by IO, 1980–2015. Y-axes are adjusted to each IO’s maximum

Intergovernmental policy is also asymmetrically distributed over time. In Fig. 2, we plot the annual count of policy acts for the IOs in our sample between 1980 and 2015. For some IOs, including ASEAN and AU, we note patterns of near-consistent growth in policy output.^15^ For example, the AU’s average annual output increased from 13 decisions in the 1980s to 80 in the 2010s, possibly due to the transition to a new institutional architecture. Most IOs mix periods of growth in output with stagnating or decreasing output. For example, we note that the EU’s annual policy output in the 1980s was larger than that of the 1990s and early 2000s, before increasing again in the latter part of the 2000s. Yet another group of IOs, including the NC and COMW, exhibit no clear temporal pattern, suggesting that their output has been fairly stable, save for the natural randomness that affects any decision-making process.

While changes in policy output may have IO-specific causes (Sommerer et al., 2022), including changes in institutional design or membership, the aggregate trend of increasing output levels likely reflects the combination of increasing regional institutionalization (Acharya, 2006; Lenz, 2021), broadening policy mandates (Hooghe et al., 2019: 147) and the relaxation of Cold-War-era political constraints during the 1990s and early 2000s, particularly acute for the UN (cf. Ikenberry, 2010). These temporal patterns grant no consistent support to the notion that multilateral cooperation generally has been in a state of crisis from about 2010 onwards (Broome et al., 2015; Hale et al., 2013). While gridlock may be a reality for some IOs on some issues, the long-term overall trend appears to be one of growing policy output.

### Policy topic

**Fig. 3:**
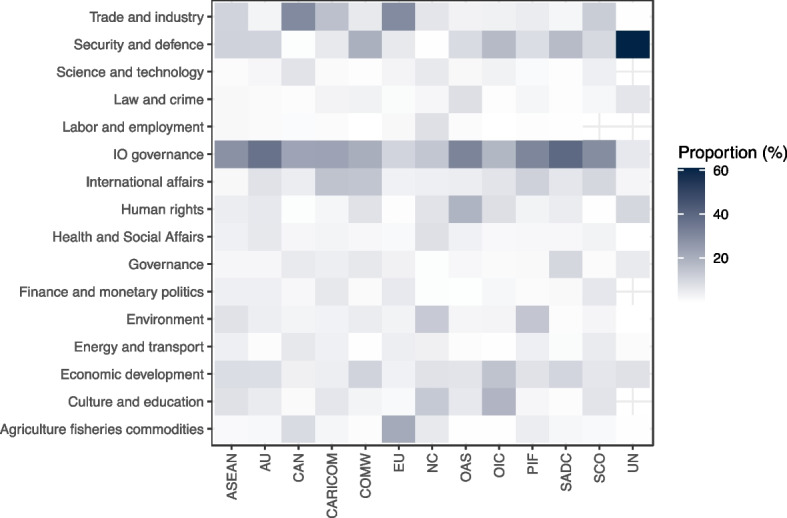
Policy topic, proportional distribution of acts for each IO in the sample. Each column (IO) adds up to 100 percent

An examination of the distribution of policy output across different topics confirms that the multi-issue mandates of these IOs are matched with broad policy agendas in practice (Fig. 3; see also Fig. A3). On average, each topic represents a mere 6.4 percent of an IO’s output (the median is 3.9 percent) and in only a handful of cases do individual topics represent a dominant portion of an IO’s total agenda. The IO with the most focused (or least dispersed) agenda in this sample is the UN, where 61 percent of the coded policy output is categorized as relating to security and defense, reflecting the unique role of the Security Council in the global management of peace and conflict.^16^ Other IOs with above-average agenda concentration are the EU and Andean Community (CAN), which both devote around 30 percent of their policy output to the topic of trade and industry. Despite these few examples, we generally find that IOs with multi-issue mandates have correspondingly broad policy agendas. This finding speaks to how cooperation may be facilitated by issue-linkages and processes of spillover (Lenz et al., 2022). Because of the more open-ended character of their mandates, these IOs more easily expand cooperation to new policy areas in response to emerging problems.

**Fig. 4:**
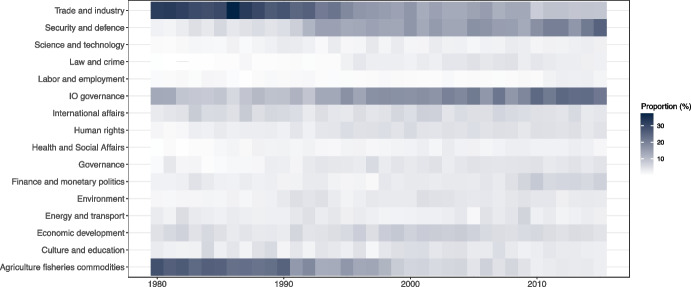
Policy topic, proportional distribution of all IO policy acts in sample, 1980–2015

Another striking pattern that emerges from these data (Fig. 3) is that a considerable portion of the policy output relates to IO governance, i.e., the administrative and operational aspects of running an organization. On average, IOs in our sample devote nearly a quarter of their policy output to such matters, and in nine IOs it represents the dominant topic. Furthermore, as a proportion of all acts in the sample, policy on IO governance has grown steadily, increasing from less than 10 percent in the late 1980s to well above 20 percent in the 2010s (Fig. 4).

This pattern points to an increasing bureaucratization of IOs: over time, IOs appear to devote a growing part of their attention to the development of their governance structures. It suggests that tendencies often associated with national political systems, such as the expansion of bureaucratic organizations (Meyer & Craig Brown, 1977; Dunleavy, 2014), prevail also among IOs. This pattern underlines the value of research on issues like IO funding, budgeting, and staffing (Goetz & Patz, 2017; Parizek, 2017; Parizek & Stephen, 2021), and their possible impact on policy-making (cf. Debre & Dijkstra, 2021a; Sommerer et al., 2022).

Other temporal trends are visible in Fig. 4. We note, for example, the increasing proportion of acts dealing with security and defense. Whereas only two IOs (the UN and the OIC) adopted acts relating to this topic in 1980, seven IOs were active in this area in 2015, and the aggregate proportion of output increased from around 2 to 25 percent in the same period. This increase suggests that the widening operational role awarded to IOs in the management of security challenges, in particular relating to civil war (Fearon, 2017; Tavares, 2009), is underpinned by a robust increase in related policy decisions. It may also signal the increasing securitization of traditionally non-security domains, like health, climate, or energy, which have become important topics in IO security policy deliberations (e.g., Kelle, 2007).

Taken together, these patterns signify shifts in the attention of multi-issue IOs that would have been impossible to demonstrate in the absence of broad, comparative data on multi-issue IOs. With this variable alone, we are able to point to patterns that suggest IOs are becoming more active, cover a wider set of issues, and are increasingly concerned with their own administration.

### Policy type

**Fig. 5:**
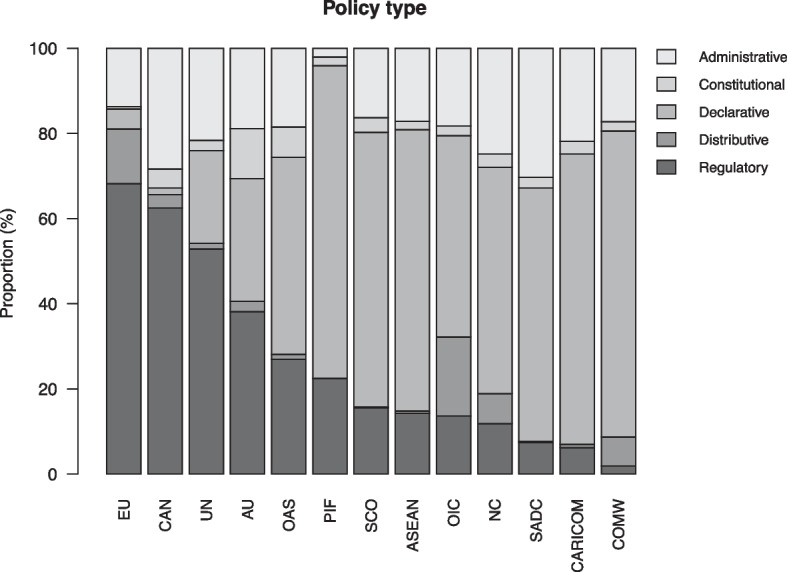
Policy type, proportional distribution of acts for each IO in the sample

In Fig. 5, we disaggregate the data by policy type and IO. We note that only a few IOs – the Andean Community (CAN), the EU, and the UN – adopt mostly regulatory acts, indicating that they are unusually oriented toward using policy to address problems of collaboration or coordination. Viewed from a perspective of comparative regionalism, regional IOs do not follow the regulatory trajectory of the EU, with the exception of the CAN. While previous literature has pointed to similar findings, especially for Africa (e.g., Fioramonti & Mattheis, 2016), our comprehensive policy data is able to confirm it.

We also see that many IO policy acts are categorized as declaratory (medium gray in figure). On average, 47 percent of an IO’s policy acts fall into this category and in several IOs – the Pacific Islands Forum (PIF), the Commonwealth (COMW), the Caribbean Community (CARICOM), and Association of Southeast Asian Nations (ASEAN) – the number is above 65 percent. The IOs that are least likely to adopt declarative policy acts – the EU, CAN and UN – were also the most likely to adopt acts categorized as regulatory.

The third most common policy type is administrative. For most IOs in the sample, about one fifth of policy acts are categorized as administrative. Given the substantial role of IO governance in shaping administrative policies, as discussed earlier, it is not surprising that this type of policy is prevalent. This finding again underlines the relevance of recent research on international public administration (Knill & Bauer, 2016; Goetz & Patz, 2017; Parizek, 2017; Parizek & Stephen, 2021).

The other two policy types – distributive and constitutional – are considerably rarer, amounting to 4.6 and 3.6 percent of the total acts, respectively. With regard to constitutional output, this is likely a reflection of the fact that IOs only occasionally revisit their foundational rules and organizational structure. With regard to distributive output, the low proportion suggests that IOs stand in contrast to legislatures at the national level, where redistributive policy is commonplace and an integral element of taxation systems in most welfare states.

**Fig. 6:**
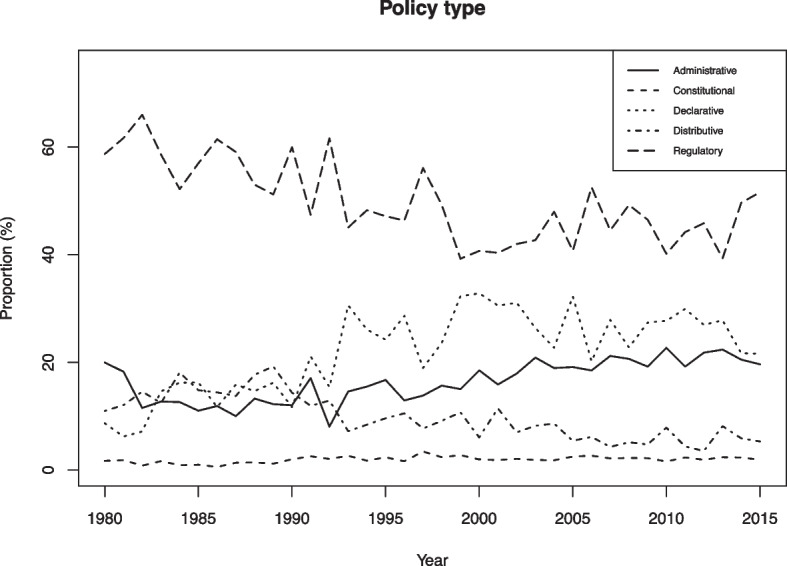
Policy type, proportional distribution of all IO policy acts in the sample, 1980–2015

As illustrated in Fig. 6, several of these patterns accentuate with time. Taken as a whole, we observe a growth in declarative and administrative policy types at the expense of the distributive type, while the regulatory and constitutional output proportions do not change dramatically. The substantive proportion devoted to declarative policy suggest that IOs continue to seek to influence actors via non-regulatory discursive means. This pattern is consistent with research showing that IOs exhibit a growing propensity to engage in verbal strategies such as “naming and shaming” (Squatrito et al., 2019). If the EU is excluded (Fig. A5), the data suggest that IOs are increasingly occupied with regulation, while there is a continued large share of declarative and administrative output. The rise in the proportion of regulatory output when the EU is excluded suggests that other IOs over time are moving in the direction of the type of regulatory governance that has characterized the EU for a long time.

### Policy instrument

**Fig. 7:**
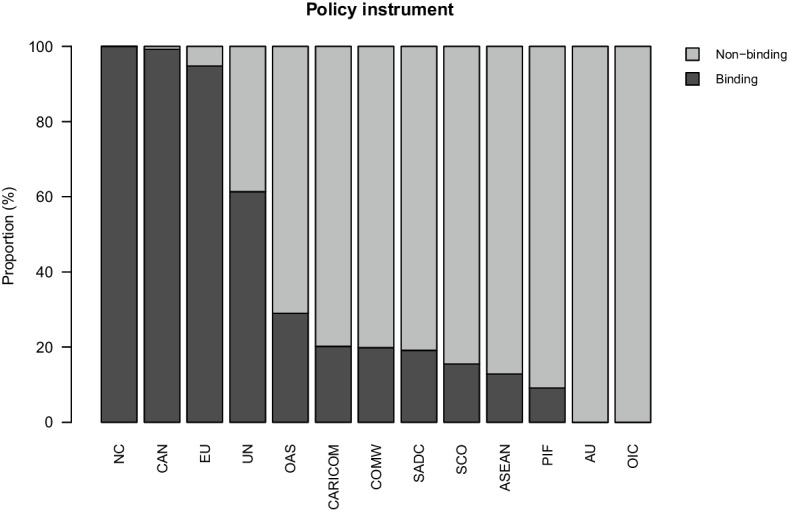
Policy instrument, proportional distribution of acts for each IO in the sample

Figure 7 presents IO-level data for policy instrument, showing the proportion of each IO’s policy output that has binding and non-binding properties, respectively. The overarching pattern is that there are two categories of IOs. The first consists of IOs that rely on both types of instruments, some binding and some non-binding. While the precise breakdown varies between organizations, we would place ASEAN, CARICOM, COMW, OAS, PIF, SADC, SCO, and UN in this category. The second category consists of IOs whose policy output is dominated by one policy instrument, producing either “hard” or “soft” law but not the other. Here, the AU and the OIC produce mainly non-binding output, whereas CAN, NC, and EU rely primarily on binding agreements. To a large extent, these patterns of variation are determined by the legal instruments available to IOs, as stipulated in their founding treaties. Nonetheless, this finding suggests additional need to understand what underlies why some IOs rely on binding policy instruments and other less so.

**Fig. 8:**
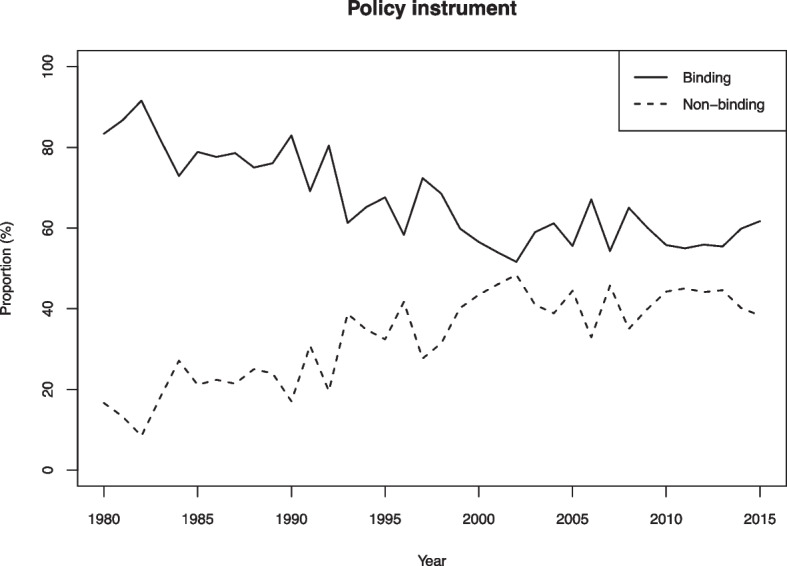
Policy instrument, proportional distribution of all IO policy acts in the sample, 1980–2015

If we examine policy instrument from a longitudinal lens, as we do in Fig. 8, we note that the aggregated data are marked by a trend toward more non-binding output. Whereas more than 80 percent of policy acts were categorized as binding in the 1980s, this dropped to 52 percent in the period since 2000. With only two categories, it is easy to see that the share of non-binding output increased from 20 to 48 percent during the same period. If the EU is excluded (Fig. A6), the temporal trend toward an increasing proportion of non-binding output remains. While we should be cautious not to draw conclusions about the legal implications of specific policies, which must be studied at the act-level, this pattern provides an empirical basis to evaluate patterns of international legalization (Alter, 2014; Goldstein et al., 2001).^17^ Indeed, the data suggest that legalization is driven in particular by non-binding policy output. This pattern aligns well with the above observations that IO policy output is becoming increasingly declaratory and less regulatory. While much scholarly focus has been on hard law and its connection to legalization, the IPOD data suggest that greater attention to soft law is merited, as it represents a significant portion of intergovernmental policy.

### Policy target

**Fig. 9:**
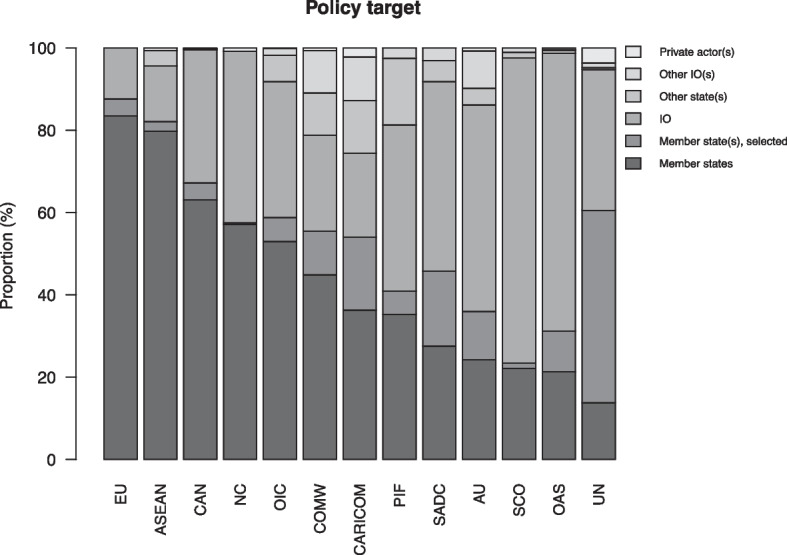
Policy target, proportional distribution of acts for each IO in the sample

**Fig. 10:**
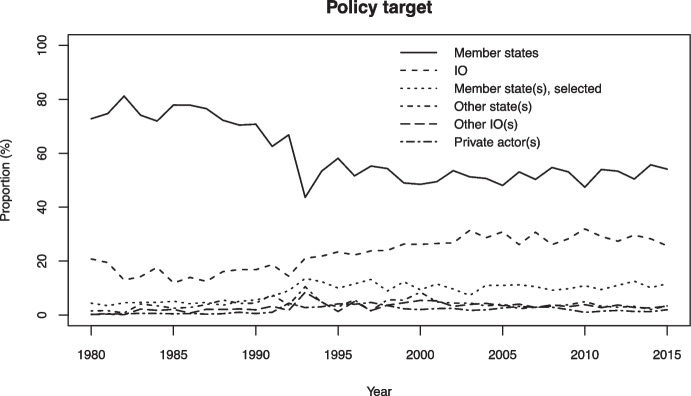
Policy target, proportional distribution of all IO policy acts in the sample, 1980–2015

Figures 9 and 10 illustrate patterns in policy target. It is clear from Fig. 9 that the most common target of intergovernmental policy are member states, addressed either as a collective (58 percent of all acts) or as individual states (9 percent), which is in line with most conventional theories on the role and function of IOs (Martin & Simmons, 2012). The three IOs that partly deviate from this pattern – the AU, the OAS, and the SCO – all disproportionately target the IO’s bureaucracy, which is the second most common target in the data as a whole. This pattern aligns well with the above observations regarding the commonality of administrative policy relating to IO governance.

Only about 10 percent of all policy acts relate to actors other than IO member states and IO bureaucracies. Of the IOs that commonly target external actors, only the UN routinely targets private actors. About one UN policy act in seven is addressed to such actors. A likely interpretation is that this reflects the UN’s involvement in intrastate armed conflict, where it conventionally seeks to manage disputes between governments and non-state rebel groups. For example, if a policy act sets out the mandate of a peacekeeping operation that seeks to monitor a ceasefire between a government and a rebel group, the latter is categorized as a non-state target in our data.

More generally, the finding that IOs only rarely target non-state actors provides nuance to research that examines IO relations with such actors. For example, it may suggest that transnational public–private governance initiatives (Westerwinter, 2021) or orchestration initiatives (Abbott et al., 2015) only rarely are based on intergovernmental policy. It may also indicate that IO policies which ultimately are aimed at regulating the behavior of non-state actors are formally targeted at states, who are expected to set up systems for enforcing these policies vis-à-vis domestic non-state actors. While certain policies targeting non-state actors may have increased with time, such as those intended to shame non-state actors (Squatrito et al., 2019) or to facilitate their involvement with IOs (Tallberg et al., 2013), our findings here suggest that a relatively small portion of IO policies target non-state actors.

If we look at the distribution of policy target over time, as we do in Fig. 10, we note two trends within the dominant target categories. First, IO policy increasingly targets IOs themselves, a category that grew from an average of around 15 percent in the 1980s to 25–30 percent in the post-2000 period. This increase is partly, but not exclusively, due to the developments in the EU (see Fig. A7) and generally aligns with our previous finding that IO governance is an important aspect of intergovernmental policy. Second, while IOs predominantly target member states, they have become increasingly willing to single out specific member states, which corresponds with other research showing that IOs name and shame their members (Squatrito et al., 2019). This category represented less than 5 percent of all IO acts in the 1980s but increased to about 10 percent in the 1990s and remained at that level until the end of the observation period.

## Illustration: Institutional friction and change in IO policy agendas

In this section, we present a concise example of how the IPOD dataset can be used to provide new insights into IO policy-making and responsiveness to a changing environment. More specifically, we investigate macro dynamics of IO policy agendas, where agendas are understood as the allocation of output across different policy topics each year. When and how does this allocation change? When agendas change, does it happen incrementally or in sudden and disruptive breaks with the status quo? A deeper understanding of the broader patterns of IO policy-making – and the factors that expedite or impede agenda adjustment – can provide insights into whether and how these organizations respond and adapt to their political context.

We approach these questions from the perspective of punctuated equilibrium theory (PET), as this has been developed in the comparative agendas literature (Baumgartner & Jones, 2010; Jones & Baumgartner, 2005).^18^ The key proposition of PET is that political attention is limited and institutions are marred by friction, leading to policy agendas that are marked by periods of stability with little or no change, which are interrupted by sudden punctuations, when agendas change dramatically in a short period of time. This dynamic of punctuated equilibria has been established in several studies on American public policy (Baumgartner & Jones, 2010; Jones & Baumgartner, 2005), comparative public policy (Baumgartner et al., 2009), and individual countries and policy contexts (e.g.,Breunig & Koski, 2006; Jennings et al., 2020). The typical argument is that less punctuated agendas reflect political institutions that operate more seamlessly and are therefore more responsive to ongoing changes in preferences and emerging issues. As such, PET is one way to approach specific facets of IO performance (Gutner & Thompson, 2010; Lundgren et al., 2018; Tallberg et al., 2016).

Since IPOD contains comparable data on many IOs across a long period of time, it provides a good opportunity to carry out a robust evaluation of PET at the international level. With the exception of studies focused on the EU (Alexandrova et al., 2016) and small samples of IOs (Lundgren et al., 2018) – both of which are inspirations for our illustration here – there are no systematic evaluations of PET at the international level. To fill this gap, we assess whether IO policy agendas exhibit characteristics typically associated with punctuations: more cases of no or little change, combined with more cases of extreme changes. Investigating the degree of punctuation can provide insights into how responsive IOs are to changes in their environment and whether institutional features explain variation in responsiveness.

We follow the conventional approach in the PET literature and examine the characteristics of frequency plots of year-on-year changes in agenda attention (Baumgartner et al., 2009; Lundgren et al., 2018; see also Padgett, 1980 for technical details). In a policy process without friction, we would expect a distribution of year-on-year changes to overlap with the normal curve, as policy responds flexibly to changes in the state of the world. In contrast, for a policy process characterized by institutional friction and punctuation, we would expect a distribution that is leptokurtic; that is, a distribution with thicker tails (reflecting instances of extreme change) and tall central peaks (reflecting periods of little or no change).

**Fig. 11:**
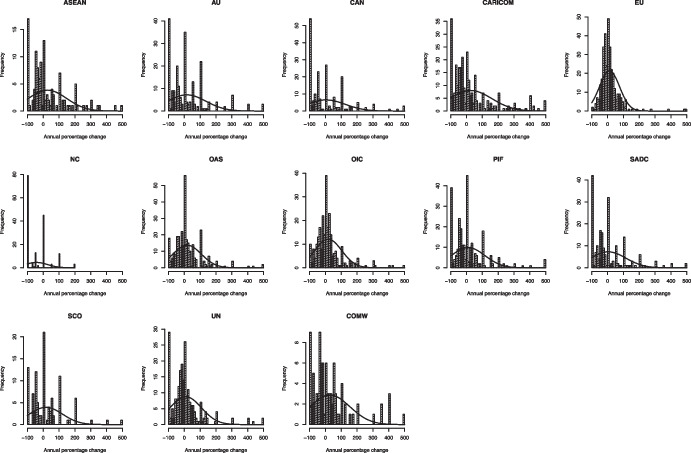
Year-on-year percentage agenda changes, 13 IOs, 1980–2015

Figure 11 exhibits the year-on-year change (Δ) distributions for the 13 IOs in our data.^19^ Visual inspection suggests that the majority of IOs have policy agendas that match the characteristics predicted by PET. We observe many observations at or around zero, indicating years in which the agenda space given to any given topic remained in equilibrium. However, we also observe that there are many observations in the tails, indicating dramatic year-on-year changes in the attention to particular issues.^20^ These are punctuations of the agenda equilibrium – periods when a topic emerges anew or receives increasing attention, or when a topic wanes or drops off the agenda completely. For example, a change value of + 100 percent indicates a doubling of attention whereas a score of -100 percent corresponds to a topic falling off the agenda completely. The observed patterns of slender peaks and extended, thicker tails suggest that the distributions are leptokurtic and indicate agendas dynamics that are consistent with PET.

We note that the IO policy agendas in our sample exhibit varying degrees of punctuation. This suggests that some IOs more seamlessly reallocate attention across topics, updating their policy agendas in response to a changing environment, while other IOs reallocate attention more rarely, leading to agendas marked by more sudden and dramatic shifts. For example, the agendas of the EU and the UN are more punctuated than those of the CAN or ASEAN. Comparative analyses of agenda punctuation have sought to explain such patterns by pointing to variation in institutional friction (Baumgartner et al., 2009; Baumgartner et al., 2009; Lundgren et al., 2018). The expectation is that systems with greater hurdles to policy-making, due to divisions of power and procedural barriers, have higher institutional friction and more punctuated agendas than systems where such hurdles are less demanding.

To examine whether institutional friction can explain variation in the degree of agenda punctuation across the IOs in our sample, we follow the approach of Baumgartner et al. (2009) and Lundgren et al. (2018) and examine whether institutional features correlate with statistical measures of agenda punctuation. Drawing on rational choice models of collective decision-making (Scharpf, 1997; Lake & Powell, 1999; Tsebelis, 2002), Lundgren et al. (2018) identifies institutional friction in IO policy-making as a function of decision rules, membership size, and preference heterogeneity. We extend this model to our larger sample and measure and rank each IO on these variables to produce an additive institutional friction index (see Table A3 for details).

*Decision rule* is operationalized as an IO’s mean pooling score, drawing on MIA data (Hooghe et al., 2017). Higher degrees of pooling entail a greater extent of majoritarian decision-making and consequently lower institutional friction. *Membership size* is the count of IO member states (Pevehouse et al., 2020). All else equal, a higher number of member states means higher transaction costs in decision-making. *Preference heterogeneity* is operationalized based on a version of the composite measure proposed in Lundgren et al. (2018), capturing the variance among an IO’s membership in income level, economic size, political regime, and cultural composition (Table A3).

The institutional friction index is the cumulative rank of each IO along these three dimensions (Table A3). IOs with less restrictive decision rules, smaller memberships, and more homogeneous preferences receive lower scores, indicating lower institutional friction; IOs with taxing decision rules, larger memberships, and more heterogenous preferences receive higher scores, indicating greater institutional friction.

**Fig. 12:**
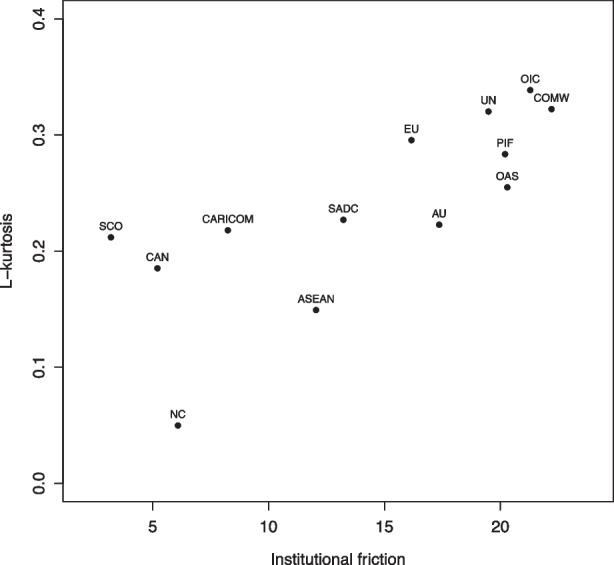
Institutional friction and degree of punctuation (L-kurtosis) in IO policy agendas

Figure 12 illustrates the relationship between institutional friction and leptokurtosis in our sample of IOs. The correlation is positive and statistically significant (Pearson’s r = 0.73; p < 0.01), indicating that higher degrees of institutional friction are associated with more punctuated agendas.^21^ The implication is that policy-making in IOs with higher levels of institutional friction are more punctuated, reflected in a combination of inertia and dramatic reallocations of agenda attention, whereas policy-making in IOs with lower levels of institutional friction evolves more smoothly.

These findings generalize the results in Lundgren et al. (2018) to a wider and more diverse sample of IOs. Where Lundgren et al. (2018) studied five IOs using automatized dictionary methods, we study a sample of 13 IOs and employ more nuanced, hand-coded data. This shows that PET, as developed in the comparatively agendas literature, has applicability in the domain of international organizations. While this argument was rather tentative in Lundgren et al. (2018) and only narrowly examined in Alexandrova et al. (2016), our application here provides a more robust corroboration.

More generally, this empirical illustration demonstrates one way in which systematic and comparative data on IO policy can be used to detect wider patterns in IO policy-making. When combined with theoretical tools developed in the policy agenda literature, such patterns can also provide insights into the factors and actors that shape IO agenda-setting (e.g., Princen, 2011) and institutional performance (Gutner & Thompson, 2010; Tallberg et al., 2016). Crucially, in contrast to the majority of the literature on these topics, the data presented here would allow researchers to engage with these questions from a comparative perspective.

## Conclusion

In this article, we have presented a novel dataset on the policy output of IOs. While the study of international organizations has become increasingly comparative, existing data resources on the policy output of IOs have remained focused on single organizations or policy fields. IPOD represents an effort to overcome this gap, offering data on the policy output of 13 multi-issue IOs in the period 1980–2015, covering close to 37,000 individual policy acts. In this article, we have described the construction and coverage of this dataset, identified key temporal and cross-sectional patterns in the data, and offered an illustration of how the data may be used to study policy agenda dynamics. By way of conclusion, we summarize three main ways in which this dataset may be useful in future research on global governance and reflect on ways in which it may be further extended.

First, IPOD generates *descriptive insights* that improve our understanding of the functioning of IOs and that speak to ongoing debates in the study of global governance. It provides researchers with a fine-grained perspective on the structure of IO policy output in terms of volume, topic, type, instrument, and target, making it possible to identify larger patterns and trends across time, policy areas, and organizations. As revealed in this article, several of those patterns and trends have implications for established expectations in existing research. For instance, while some suggest that IOs are in decline (Debre & Dijkstra, 2021b), our data reveal that those organizations which persist are producing more output over time, across a broader range of issues, and targeting a larger set of actors, indicating a deepening and widening of IO authority (Zürn, 2018). Likewise, we find that IOs devote a significant – and increasing – part of their policy output to self-governance, pointing to a growing bureaucratization of global governance and the value of research on issues of international public administration, such as funding and staffing (Goetz & Patz, 2017; Parizek & Stephen, 2021). In a similar fashion, the data show that policy output is increasingly declaratory and often nonbinding, which speaks to research on legalization in international cooperation (Abbott & Snidal, 2000; Goldstein et al., 2001) and suggests that soft law may have a more prominent role than previously understood.

Second, this dataset invites explorations of IO policy output as a *dependent variable*. Policy output varies across IOs in intriguing ways that call for explanation. Why are some IOs able to produce impressive levels of policy output while others are deadlocked? How come some IOs primarily produce regulatory policy, while others engage in declaratory policy? And what accounts for variation across IOs in terms of the bindingness of policy output? While advances in the explanatory study of IO policy have been held back by the lack of comparative data, IPOD offers new possibilities. Our illustration of how institutional friction contributes to variation across organizations in the punctuation of policy agendas is suggestive of the potential to contribute to explanatory research on IO policy output (see also Lundgren et al., 2018). Scholarship in international relations suggests a range of factors that may be helpful for explaining patterns in IO policy output, such as state preferences, power asymmetries, norm dynamics, and institutional designs.

Third, IPOD may be used for analyses where IO policy output is a relevant *independent variable*. For instance, in studies of regime effectiveness, policy is typically seen as the first step of three in a process leading from output (policy) to outcome (compliance) and impact (problem-solving) (Young, 2014). Likewise, research on the performance of IOs usually conceives of policy output as a necessary condition for an organization to reach the goals it was set up to achieve (Gutner & Thompson, 2010; Lall, 2017). Other areas of inquiry for which data on policy output may prove useful include research on the legitimacy of IOs, the distributive consequences of IOs (Fehl & Freistein, 2020), and the diffusion of policies across IOs (Sommerer & Tallberg, 2019). With the new data offered by IPOD we can thus better understand the linkages between the policies IOs produce and these broader phenomena.

While IPOD provides new opportunities for research on a variety of issues, one additional avenue for future work is to further extend the dataset. While ambitious, the dataset is based on a limited sample of IOs. Though our sample is representative of the population of multi-issue IOs, it excludes task-specific IOs. This leaves open future avenues for further comparison of international policy between multi-issue and task-specific IOs that produce policy output. The approach advanced here suggests a conceptual framework and a set of operationalizations that should be of value for studying any type of international organization, including those focused on more narrow policy domains. Extensions of our data to include additional IOs or existing IOs over a longer time frame would offer insights into the generalizability of the patterns we have observed. In addition, there are opportunities to extend the data collection to types of IO output other than intergovernmental policy, such as the output of supranational bureaucracies and courts. Such extensions would further strengthen the usefulness of this dataset. Yet already in its present form, IPOD helps to close a significant data gap in the comparative study of international organizations and reveals intriguing patterns in IO policy with direct implications for current debates on global governance.

## Supplementary Information

Below is the link to the electronic supplementary material.

Supplementary file1 (PDF 728 KB)

Supplementary file2 (ZIP 1.46 MB)

## Data Availability

The data analyzed in this article and replication code will be made publicly available in the Harvard Dataverse depository upon publication.

## Footnotes

1 There are also datasets on policy-specific agreements struck outside formal IOs, such as datasets on preferential trade agreements (Dür et al., 2014), bilateral investment treaties (Haftel & Thompson, 2018), and international environmental agreements (Mitchell, 2003).

2 Similarly, datasets focused on decision-making (such as the DEU datasets) privilege information on process and negotiation positions over information on the design and orientation of the considered policies.

3 Coding of the issue areas for IOs in the COW-IGO database was based on the full name and the self-description on the official homepage.

4 In contrast with the COW-IGO data, the MIA dataset includes information on the issue area. All MIA IOs with core mandates in at least three issue areas when coding began in 2017 are included in our definition of multi-issue IOs in COW-IGO, demonstrating a high degree of overlap between the two approaches. Three of our selected IOs had fewer issue areas in their core mandate (but not their overall policy scope) according to MIA for some years during our time frame: SADC (1980–1992), COMW (1980–1990), OIC (1980–1981).

5 We recognize that several important multi-issue IOs are left outside of our sample – such as Mercosur and ECOWAS – which deserve consideration for inclusion in future data efforts.

6 The United Nations has two principal intergovernmental decision-making bodies, the Security Council and the General Assembly. We have decided to focus on the Security Council, for three main reasons. First, as per the UN Charter, it has more substantive powers than the General Assembly. Second, the Security Council plays an important role in the strategic governance of the UN, including by deciding on new members and making recommendations for the appointment of its top official, the Secretary General. Third, the Security Council has gradually widened its policy scope, such that it increasingly considers issues of human rights, development, environment, and gender, providing a bridge between these issue areas and its traditional domain of security.

7 Note that some IO decision-making bodies have “gap years” where they either did not meet or did not produce any policy output.

8 Nordic Council, B/181, 1998,”Förslag till ny överenskommelse om Nordiska Miljöfinansieringsbolaget, NEFCO”.

9 Organization of Islamic Cooperation, Resolution 36/12, 1981, “Assistance to the Republic of Guinea”.

10 UN Security Council, Presidential Statement S/PRST/2011/9, 2011.

11 Caribbean Community, “Communique Issued at the Conclusion of the Sixth Meeting”, para 14, 1985.

12 Southern African Development Community, Communiqué, Saint Baie summit, August 2004, para 44.

13 Articles 24 and 25 of the UN Charter obligate member states to comply with the decisions of the Security Council. There is some debate about whether Presidential Statements can generate legal obligations, but the prevailing consensus is that they do not (cf. Gray, 2018a, b).

14 Figures A3–A6 in the online appendix present aggregate patterns for our sample of IOs, excluding the EU. We note in the text when a particular pattern is influenced by the inclusion or exclusion of the EU.

15 These and all other temporal trends identified in the descriptive analysis are statistically significant at p < 0.05, both in linear regression models and Mann–Kendall tests using bootstrapping to adjust for serial correlation. Differences of means discussed in the text are statistically significant at p < 0.05.

16 Collecting data on the UN General Assembly (UNGA) would allow scholars to assess the distribution for that body. Our expectation is that the UNGA would show a less concentrated topic profile.

17 The trend remains even if we account for growing overall IO policy output. The annual average of binding IO policy acts was lower in the 2010s than in the 1980s.

18 In political science, Krasner (1984) contributed to popularizing the term of punctuated equilibrium, when discussing how state structures undergo periods of both institutional change and stasis. PET builds on the same analogy to offer a refined theory of change and stability in policy agendas specifically.

19 Table A3 in the online appendix presents distributional statistics, including the L-kurtosis values for each IO-specific distribution.

20 Following conventions in the literature, the distributions are truncated at -100 percent and + 500 percent.

21 Regressing leptokurtosis scores on institutional friction yields a linear model, *y* = 0.08 + 0.009*x*, where *y* is leptokurtosis and *x* institutional friction. The coefficient for *x* is statistically significant at the p < 0.01 level.
