# Supplementary material for: Introducing the Intergovernmental Policy Output Dataset (IPOD)
Source: Rev Int Organ. 2023 Jun 6:1–30. Online ahead of print. doi: 10.1007/s11558-023-09492-6 (PMC10242228; doi:10.1007/s11558-023-09492-6)
Supplement: Supplementary file 1 — Supplementary file1 (PDF 728 KB) [file 11558_2023_9492_MOESM1_ESM.pdf]

Online appendix

Introducing the Intergovernmental Policy Output Dataset (IPOD)

Figure A1. Geographical scope of IPOD sample of IOs.

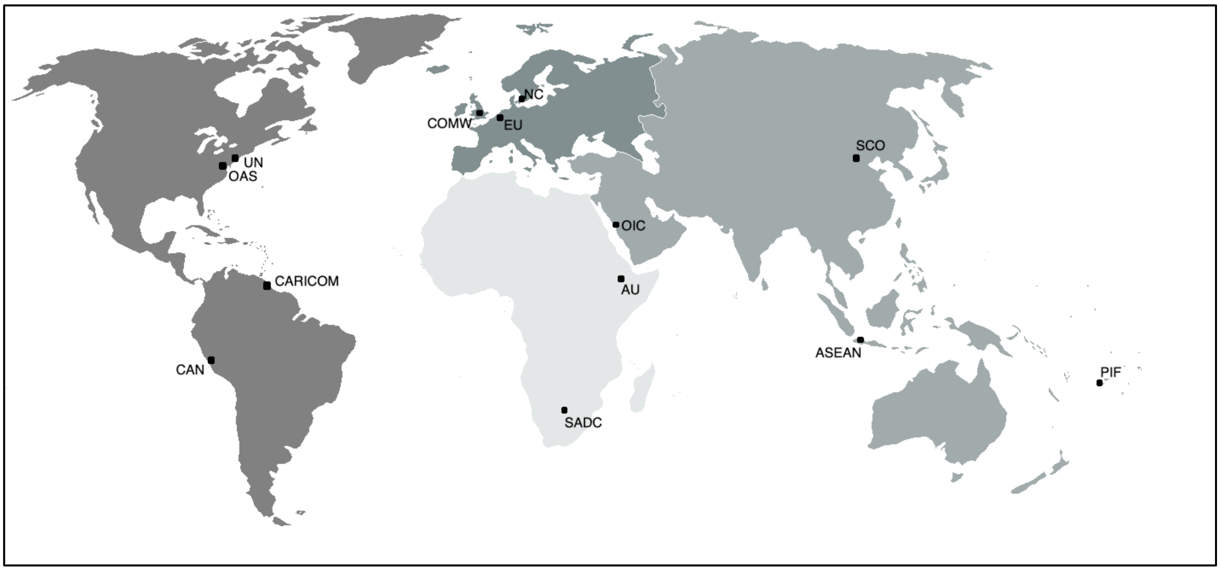

Table A1. Multi-issue IOs: IPOD Sample vs. COW-IGO Population

Africa	Americas	Asia-Pacific	Europe	Global
**OAU/AU**	**CAN**	**ASEAN**	**EU**	**COMW**
**SADC**	**CARICOM**	**SCO**	**NC**	**OIC**
AMU	**OAS**	**PIF**	ArcticC	**UN**
CEMAC	ACS	ASEF	BC	APEC
CEPGL	AmCC	BSEC	BENELUX	D8
COMESA	ArcticC	CIS	BSEC	NAM
EAC	LAIA	EAEC	CBSS	OECD
ECCAS	MERCOSUR	ECO	CEI	OIF
ECOWAS	OECS	GCC	RCC	OSCE
IGAD	RIO group	LOAS		UM
LOAS	SELA	SAARC		
MRU	SICA	SPC		
SACU				

Note: List of 53 multi-issue IOs active in 2015 from the COW-IGO dataset v3.0 (Pevehouse et al. 2020. Bolded IOs included in IPOD. For IO acronyms see COW-IGO codebook). An IO defined as multi-issue is active in more than two issue areas. Global IOs have member states from three or more world regions. IOs with member states from two world regions appear twice (ArcticC. BSEC, LOAS).

Table A2. Composition of membership size and institutionalization, IPOD sample

		Membership size		
		**Small**	**Medium**	**Large**
**Institutionalization**	**Weak**	AMU	**ASEAN**	**COMW**
		BENELUX	**CARICOM**	OIF
		CIS	ECCAS	**OIC**
		GCC	**PIF**	
		IGAD		
		MERCOSUR		
		**NC**		
		SAARC		
		**SCO**		
		SICA		
	**Strong**	**CAN**	COMESA	**AU**
		CEMAC	ECOWAS	**OAS**
		EAC	**EU**	**UN**
		OECS	LOAS	
			**SADC**	

Note: Data based on MIA dataset including all multi-issue IOs (n=29; three or more core issues) Bold: IPOD sample (n=13). Membership size categories: small (3-10 member states), medium (11-30 member states), and large (>30 member states); Level of authority corresponds to average of pooling and delegation score smaller/larger than 0.29 (MIA sample mean).

Figure A2. Balance of sample with MIA population of multi-issue IOs

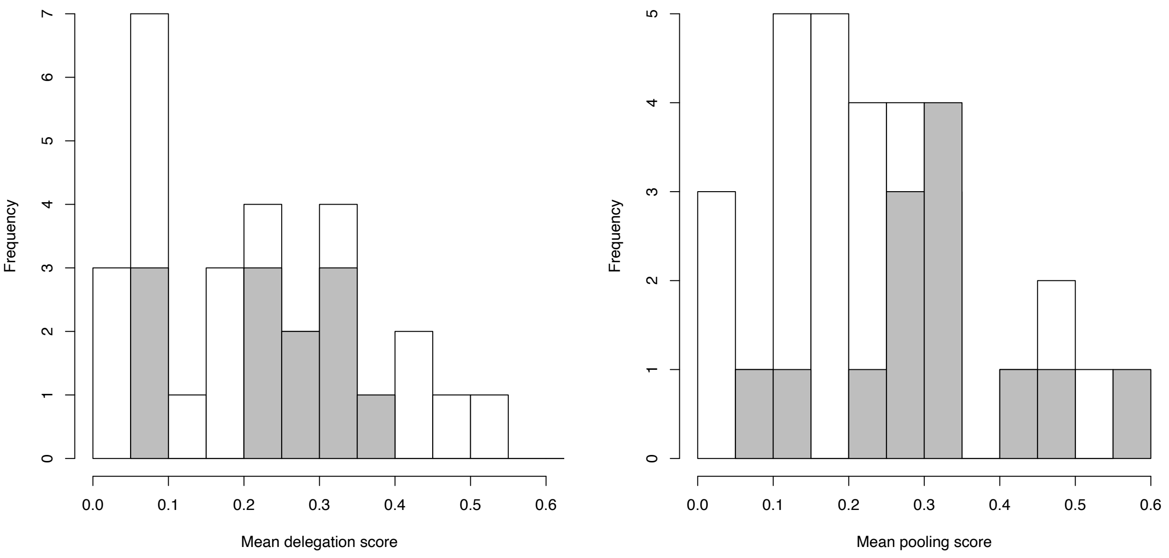

Note: Histogram of delegation and pooling values for multi-issue IOs in MIA dataset (Hooghe et al. 2017) in white. Mean values of IOs in our sample marked in grey.

Figure A3. Proportional distribution of acts across policy topics for each IO in the sample.

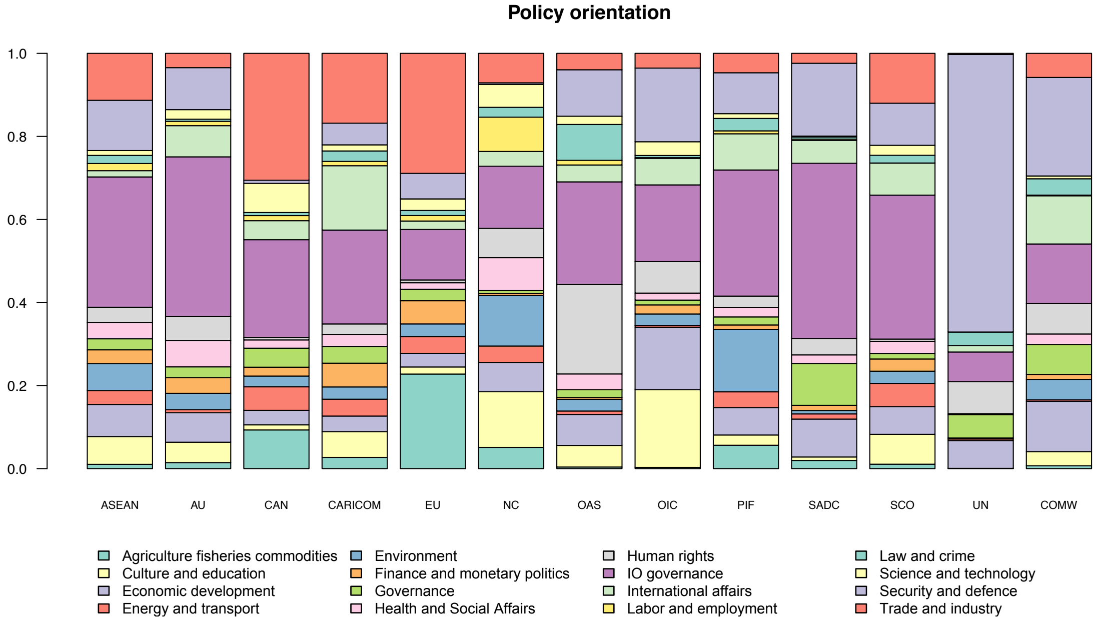

Figure A4. Policy topic, proportional distribution of IO policy acts in sample, 1980-2015 (excluding the EU).

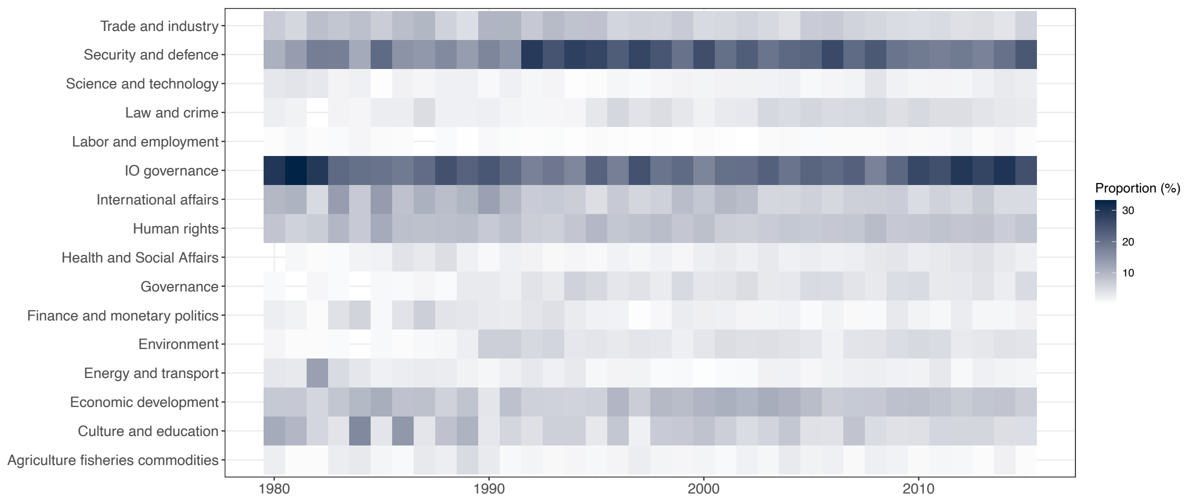

Figure A5. Policy type, proportional distribution of IO policy acts in the sample, 1980-2015 (excluding the EU).

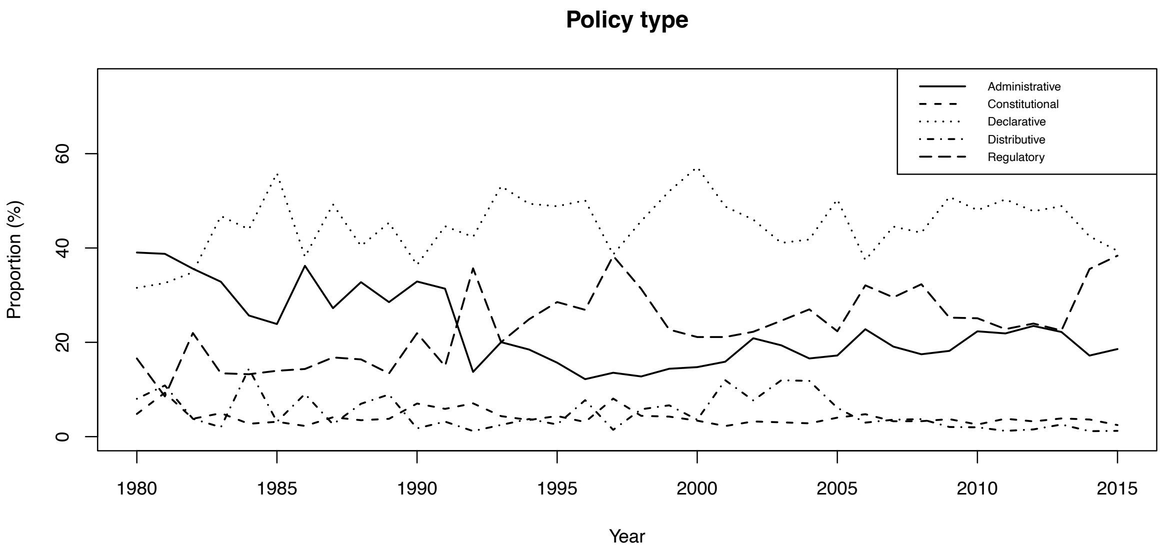

Figure A6. Policy instrument, proportional distribution of IO policy acts in the sample, 1980-2015 (excluding the EU).

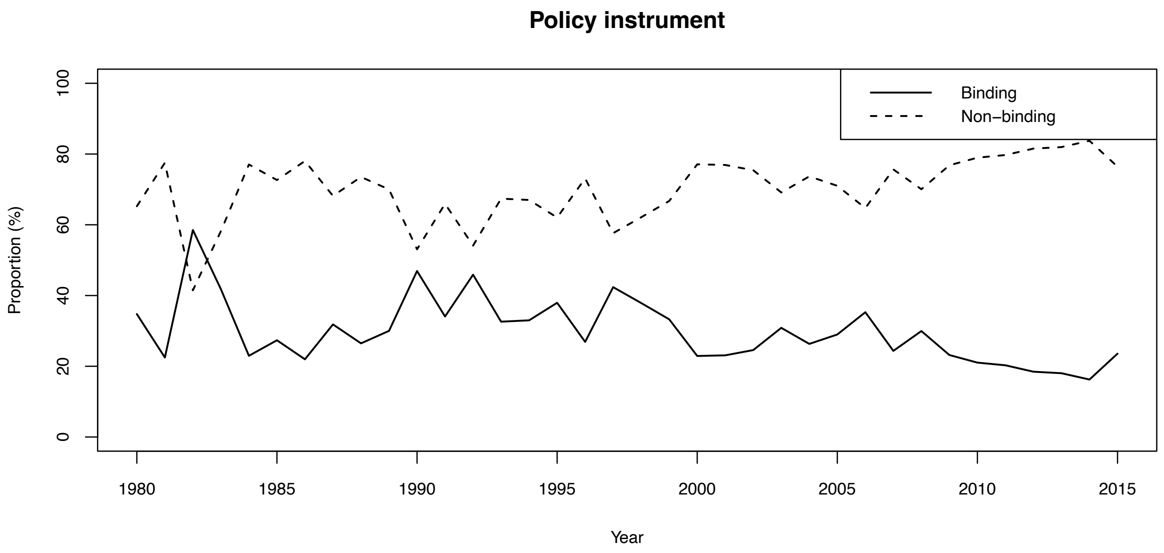

Figure A7. Policy target, proportional distribution of IO policy acts in the sample, 1980-2015 excluding the EU).

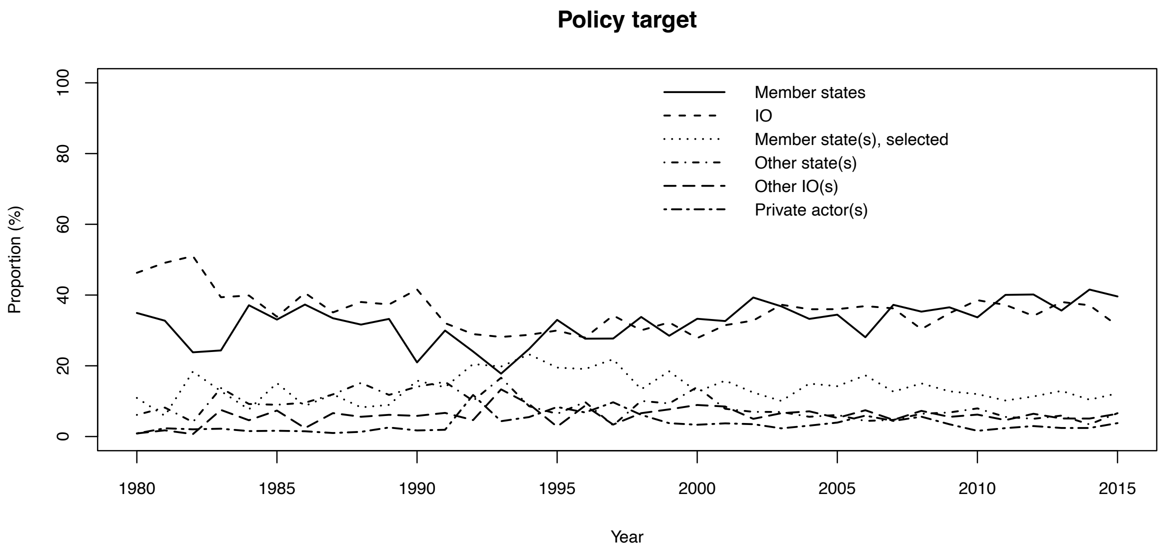

Policy target
Member states
IO
Member state(s), selected
Other state(s)
Other IO(s)
Private actor(s)
Proportion (%)
0
20
40
60
80
100
1980
1985
1990
1995
2000
2005
2010
2015
Year

Table A3. L-kurtosis and institutional friction by IO

IO	L-kurtosis of Δ distribution	Decision rules rank	Membership rank	Preference heterogeneity rank	Total institutional friction
SCO	0.21	10	2	1	3.20
CAN	0.19	8	3	2	5.20
NC	0.05	12	1	5	6.09
CARICOM	0.22	5	5	3	8.24
ASEAN	0.15	13	4	8	12.05
SADC	0.23	6	7	6	13.23
EU	0.30	11	9	7	16.16
AU	0.22	2	13	4	17.36
UN	0.32	1	6	13	19.48
PIF	0.28	9	8	12	20.20
OAS	0.25	3	10	10	20.30
OIC	0.34	4	12	9	21.29
COMW	0.32	7	11	11	22.21

Note: Total institutional friction is calculated as the mean of the rank on decision rules, membership size, and preference heterogeneity. The latter variable is calculated as the mean rank of four underlying variables: income level (real GDP/capita) (Gleditsch 2002), economic size (real GDP) (Gleditsch 2002), political regime (V-Dem liberal democracy) (Coppedge et al 2020), and ethno-linguistic fractionalization (ELF) (Fearon 2003; ranging from 0 to 1, where higher values signify greater cultural heterogeneity). We scored each IO's value based on its ordinal ranking (1–13) and aggregated the ordinal rankings across the four dimensions to reach a heterogeneity score, which was itself ranked.

Intergovernmental Policy Dataset (IPOD)

Codebook

Version: 17 March 2022

Variable	Description
IO	Name of international organization.
IO id	A unique IO id number based on COW IO id numbers (Pevehouse et al 2020). For IOs that lack COW id numbers, one will be assigned. See appendix 1 for COW IO id numbers.
Year	The year of observation.
Act id	A unique id number given to policy act. Coded based on IO id, year, and output number. Coded XXXYYYYZZZZ, where XXX is the IO id; YYYY is the year of observation; and ZZZZ is the assigned output number, starting with 1001 in each year.
Act type	The type of output, as per IO's own categorization. E.g., "resolution".
IO act number	The reference number given to this policy act by the IO. E.g., "A/58/120".
Act title	Title of act.

Policy topic

Policy topic refers to the "issue" or "subject" of a given policy act. Based on a reading of the text, each act is assigned 1-3 topic codes from the list (101-1699). The assignment of topics should be restrictive: if there is not more than 1 dominant topic, only 1 should be assigned. If a topic is frequent, but no relevant topic code exists, a new one can be created. For each main code, there is a "general or other" sub-code, which is used if the output covers an issue in general terms or in those rare instances where a topic code does not exist or a new category does not make sense.

Main code	Topic	Sub-code
**100**	IO governance	101. Budgets<br>102. Appointments<br>103. Staffing (Pensions, salaries, and benefits)<br>104. Audits<br>105. Meeting governance<br>106. Operational<br>107. IO premises and offices<br>108. IO-IO cooperation<br>109. TNA access and cooperation

		110. Organizational development (membership, institutional architecture, rules of procedure, etc.)<br>111. Appreciation and recognition<br>112. Implementation encouragement<br>199. General or other
**200**	Economic development	201. Poverty reduction<br>202. Foreign aid<br>203. Good governance promotion<br>204. Housing and Settlement<br>205. Hunger and malnutrition<br>206. Industrial development<br>207. Population and development<br>208. Disaster relief and humanitarian action<br>209. Rural development<br>210. Urban development and planning<br>211. Water, sanitation and hygiene<br>299. General or other
**300**	Health and Social Affairs	301. Infants and children<br>302. Public health<br>303. Disease prevention, treatment, immunization<br>304. Drug abuse<br>305. Drug industry and clinical labs<br>306. Nutrition<br>307. Migration<br>308. Rural and Urban housing<br>309. Welfare<br>399. General or other
**400**	Human rights	401. Children's rights<br>402. Ethnic minorities and racial issues<br>403. Freedom of speech, voting rights, right to privacy<br>404. Access to information<br>405. Gender equality and gender-based violence<br>406. Women's rights<br>407. LGBT rights<br>408. Indigenous peoples<br>409. Refugees<br>410. Religious, age, handicap-based discrimination and violence<br>411. War crimes, crimes against humanity<br>412. Torture and cruel punishments<br>499. General or other
**500**	Culture and education	501. Elementary and secondary education<br>502. Higher education<br>503. Vocational education<br>504. Research and knowledge management<br>505. Cultural heritage<br>599. General or other
**600**	Labor and employment	601. Employee relations and labor unions<br>602. Employment benefits<br>603. Labor markets<br>604. Labor standards and labor law<br>605. Migrant workers<br>606. Worker safety and protection<br>607. Youth employment and child labor<br>699. General or other

**700**	Law and crime	701. Bribery and Corruption<br>702. Crime against children<br>703. Crime prevention<br>704. Drug production, trafficking<br>705. Human trafficking<br>706. Organized crime<br>707. Police, weapon control, prisons<br>708. Rule of law<br>709. Terrorism<br>710. Transitional justice<br>799. General or other
**800**	Governance	801. Bureaucratic oversight<br>802. Non-governmental actors<br>803. Public governance and management<br>804. Regulatory reform<br>805. Risk governance<br>806. Statistics<br>807. Tax administration<br>808. Support for good governance and democratic processes<br>899. General or other
**900**	Environment and natural resource management	901. Air pollution<br>902. Chemical safety<br>903. Climate change<br>904. Consumption<br>905. Disaster risk reduction<br>906. Environmental risk management<br>907. Hazardous waste<br>908. Land conservation<br>909. Natural resource and forest management<br>910. Noise pollution<br>911. Species, plants protection, biodiversity<br>912. Sustainable development<br>913. Waste and recycling<br>914. Water safety, supply, pollution, conservation<br>999. General or other
**1000**	Agriculture, fisheries, commodities	1001. Agricultural development<br>1002. Agricultural subsidies<br>1003. Animal and crop diseases<br>1004. Animal production<br>1005. Commodity regulation<br>1006. Fisheries and Fishing<br>1007. Food inspection and safety<br>1099. General or other
**1100**	Energy and transport	1101. Airport, airlines, air traffic control and safety<br>1102. Alternative energy<br>1103. Fossil energy<br>1104. Maritime issues<br>1105. Nuclear energy<br>1106. Railroad transportation and safety<br>1107. Road traffic, road safety, highway construction<br>1199. General or other
**1200**	Science and technology	1201. Biotechnology<br>1202. Communications<br>1203. Computer industry and security

		1204. Digital government<br>1205. Governance of space and space exploration<br>1206. International scientific cooperation<br>1207. Internet<br>1208. Newspaper, publishing, broadcast industry<br>1209. Postal service<br>1210. Telephone and telecommunication<br>1211. Weather forecast and oceanography<br>1299. General or other
**1300**	Trade, economic integration, and industry	1301. Competition<br>1302. Consumer rights<br>1303. Corporate governance<br>1304. Corporate social responsibility<br>1305. Export promotion<br>1306. Industry and entrepreneurship<br>1307. Innovation<br>1308. Intellectual property rights<br>1309. Non-tariff measures<br>1310. Small- and medium enterprises<br>1311. Microfinance<br>1312. Tariff and import restrictions<br>1313. Trade negotiations and agreements<br>1314. Tourism<br>1399. General or other
**1400**	Finance and monetary policy	1401. Banking system and financial institution regulation<br>1402. Consumer finance<br>1403. Exchange rates<br>1404. Financial markets<br>1405. Monetary issues<br>1406. Productivity and growth<br>1407. Public finance and fiscal policy<br>1408. Securities and insurance regulation<br>1409. Taxation<br>1499. General or other
**1500**	Security and defence	1501. Defense alliances and security assistance<br>1502. Disarmament, arms control and nuclear proliferation<br>1503. Military aid and weapons sales<br>1504. Military installations and personnel<br>1505. Military intelligence<br>1506. Civil defense<br>1507. Intra-state violence and conflict<br>1508. Military interventions and operations<br>1509. Peacebuilding and peacekeeping<br>1510. Mediation and arbitration<br>1511. Terrorism<br>1512. Inter-state violence and conflict<br>1513. Economic sanctions<br>1599. General or other
**1600**	International affairs	1601. Decolonization<br>1602. Diplomats, embassies, citizens abroad, passports<br>1603. Border control<br>1604. International organizations and agreements<br>1605. International law<br>1606. Organization of international meetings<br>1699. General or other

Policy type

Policy type refers to the function of the policy. What does it seek to do?

1	**Regulatory**	Regulatory policy output specifies actions that target actors are either expected to take or refrain from, aiming to achieve desired interactions by addressing problems of coordination and collaboration.<br><br>Sub-codes:<br><br>**11. Coordination**<br>A coordination problem is one where all parties can realize mutual gains, but only by making mutually consistent decisions. There exist no incentives to defect once a policy is in place. Examples of regulatory output that target coordination problems are:<br>- Regulation of shipping lanes.<br>- ISO standards.<br><br>**12. Collaboration**<br>A collaboration problem implies that there may exist incentives to defect from a policy, i.e., it is not self-enforcing. Examples of regulatory output that targets collaboration problems are:<br>- Emission reduction protocols.<br>- Nuclear non-proliferation agreement.<br>- Peace treaties.
2	**Distributive**	Distributive policy output relates to the distribution or redistribution of goods and services among actors.<br><br>Sub-codes:<br><br>**21. Distributive**<br>Distributive policy regulates the distribution of a good that does not entail losses for any actor. A good emerges and is distributed without anyone losing anything. Examples of distributive policies:<br>- Allocation of internet domains.<br><br>**22. Redistributive**<br>Redistributive policy regulates the distribution of a good that entails losses for at least one actor. A good is taken from someone and given to someone else. Examples of redistributive policies:<br>- Agreement on aid program.<br>- Tax scheme.
3	**Declarative**	Declarative policy output asserts a joint position of the member states or the IO. It may be aspirational, assert agendas, or promote or condemn certain actions.<br><br>Sub-codes:<br><br>**31. Joint position**<br>A joint position is a policy statement that declares the IO's policy position on some issue.

		**32. Aspirational or symbolic**<br>Aspirational policy outlines future goals and general visions or ambitions of an IO. Examples of aspirational policies:<br>- UN General Assembly resolution recognizing the 70th anniversary of the end of World War II (A/RES/69/267).<br>- A UN resolution establishing development goals in a general way.<br><br>**33. Condemnation**<br>A policy that condemns a situation, event, organization or individual. Examples include:<br>- EU condemning the actions of ISIS.<br>- UN criticizing the human rights record of North Korea.<br><br>**34. Praise or celebration**<br>A policy that praises, appreciates, or celebrates a situation, event, organization or individual.
**4**	**Constitutional**	Constitutional policy output concerns the rules that govern an IO or changes to its general organizational structure.<br><br>Sub-codes:<br><br>**41. Membership**<br>Output concerns the IO membership, e.g., accession of new members or exclusion of existing members.<br><br>**42. Institutional architecture**<br>Output entails institutional creation or reform, e.g., the establishment of a new institutional body, office, or department.<br><br>**43. Rules of procedure**<br>Output concerns the modes of internal operation of the IO, e.g., adoption of new rules of procedure or changes in decision-making powers.
**5**	**Administrative**	Administrative policy output concerns internal operational and managerial concerns of an IO.<br><br>Sub-codes<br><br>**51. Budgetary**<br>Output relating to budgetary concerns.<br><br>**52. Other**
**9**	**Other**	

Policy instrument

Policy instrument refers to whether a decision is binding or not, i.e., whether it creates legal obligations on the part of the signatories or not.

1	**Binding**	The policy is legally binding (hard law), i.e., it imposes legal obligations on the parties.
2	**Non-binding**	The policy is non-binding (soft law). While it may have advisory functions, it does not impose legal obligations on the parties.
9	**Unclear**	

Policy target

Policy target refers to the actor at the receiving end of a particular decision or policy.

1	**IO**	The policy targets the IO itself. This could be, for example, the Secretariat of an IO, its Secretary General, or other parts of the IO.
2	**Member state(s), selected**	The policy targets one or a few named states that are members of the IO. The name of the state(s) is put in the "comments" field.
3	**Member states**	The policy targets all member states of the IO. If no particular target is mentioned, this is a likely contender.
4	**Other state(s)**	The policy targets one or several states that are not members of the IO.
5	**Other IO(s)**	The policy targets one or several IOs other than the IO itself.
6	**Private actor(s)**	The policy targets one or several private/non-state actors.
7	**Other**	The policy targets an actor not listed in 1-5.
9	**Unclear**	
